# N‐6 methylation‐related lncRNA is potential signature in lung adenocarcinoma and influences tumor microenvironment

**DOI:** 10.1002/jcla.23951

**Published:** 2021-09-24

**Authors:** Jian Zheng, Zhuochen Zhao, Junhu Wan, Manman Guo, Yangxia Wang, Zhengwu Yang, Zhuofang Li, Liang Ming, Zhaobing Qin

**Affiliations:** ^1^ Department of Otolaryngology The First Affiliated Hospital of Zhengzhou University Henan China; ^2^ Department of Clinical Laboratory The First Affiliated Hospital of Zhengzhou University Henan China

**Keywords:** bioinformatics, lncRNA, lung adenocarcinoma, m6A, tumor microenvironment

## Abstract

**Background:**

N‐6 methylation (m6A) pushes forward an immense influence on the occurrence and development of lung adenocarcinoma (LUAD). However, the methylation on non‐coding RNA in LUAD, especially long non‐coding RNA (lncRNA), has not been received sufficient attention.

**Methods:**

Spearman correlation analysis was used to screen lncRNA correlated with m6A regulators expression from the Cancer Genome Atlas (TCGA) and Gene Expression Omnibus (GEO) repositories, respectively. Then, the least absolute shrinkage and selection operator (LASSO) was applied to build a risk signature consisting m6A‐related lncRNA. Univariate and multivariate independent prognostic analysis were applied to evaluate the performance of signature in predicting patients' survival. Next, we applied Gene Ontology (GO), Kyoto Encyclopedia of Genes and Genomes (KEGG), and gene set enrichment analysis (GSEA) to conduct pathway enrichment analysis of 3344 different expression genes (DEGs). Finally, we set up a competing endogenous RNAs (ceRNA) network to this lncRNA.

**Results:**

A total of 85 common lncRNAs were selected to acquire the components related to prognosis. The final risk signature established by LASSO regression contained 11 lncRNAs: ARHGEF26‐AS1, COLCA1, CRNDE, DLGAP1‐AS2, FENDRR, LINC00968, TMPO‐AS1, TRG‐AS1, MGC32805, RPARP‐AS1, and TBX5‐AS1. M6A‐related lncRNA risk score could predict the prognostic of LUAD and was significantly associated with clinical pathological. And in the evaluation of lung adenocarcinoma tumor microenvironment (TME) by using ESTIMATE algorithm, we found a statistically significant correlation between risk score and stromal/immune cells.

**Conclusion:**

M6A‐related lncRNA was a potential prognostic and therapy target for lung adenocarcinoma.

## Graphical abstract

In this study, we further explored the role of m6A‐related long non‐coding RNA in lung adenocarcinoma. First, we found the lncRNAs related to m6A from TCGA and GEO repositories by Pearson correlation analysis. Then, we established a risk model by using LASSO method and comprehensively expounded the potential role of m6A‐related lncRNAs in lung adenocarcinoma from the aspects of prognosis verification, clinical characteristics, tumor microenvironment, and molecular mechanism.

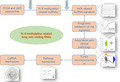

## 1 INTRODUCTION

Lung cancer is now the most common malignant tumor with its morbidity and mortality are among the first malignant tumors, which has a big threat to human life.^1^, ^2^ As important one of the histological subtypes of lung cancer, the incidence of lung adenocarcinoma has gradually surpassed that of lung squamous carcinoma in recent years and become the largest subtype of lung cancer.^3^

The pathogenesis of lung cancer is a complex mechanism in which RNA methylation plays a role in the progress.^4^, ^5^ Recent studies have showed that numerous RNA methylation modifications including N‐6 methylation (m6A), 5‐methylctisine (m5C), and pseudoguanosine are widely present in transcriptome RNA,^6^ which may be one of the pathogenesis of LUAD. With the development of next‐generation sequencing technology, m6A, the most prevalent modification of eukaryotes is gradually appearing to light,^6^, ^7^ and new localization techniques also make m6A research possible.^8^ N‐6 methylation‐related methylase and demethylase can be classified into three enzymes with respective functions that perform reversible methylation on the sixth nitrogen atoms of RNA.^9^ The way their works is shown below: enzymes called m6A “writers” (mainly including METTL3‐14, WTAP, HAKAI, WTAP, and KIAA1429) combine their subunits to form the m6A complex, which can catalyze the forward methylation of RNA.^9^, ^10^ The methylation can be reversed by demethylase, the enzymes involved in such as ALKBH5 and FTO, also known as the “erasers”.^11^ In addition, there are some reading proteins else with specific structural domains as YTH domains (YTHDC1‐3) and RBM domains (RBM15), which participate in the reading process of m6A.^12^ The m6A regulators have been shown to play a wide and profound role in the variety of human disease and to be involved in human malignant tumor via regulating RNA metabolism.^9^

Tumor microenvironment (TME) is the environment in which tumors live in and the soil for tumors grow, migrate, and invade. The interaction among stromal cells, immune cells, and tumor cells is an important factor in tumor growth and development in TME.^13^ In the progress of tumor occurrence, components of TME can be recruited by tumor cells and become the environment driving force for tumor growth and metastasis.^14^ For example, although macrophages serve as a barrier to the removal of the tumor cells, it has been reported that tumor‐related macrophages can promote tumor progression through para‐signal secretion in an existing tumor.^15^, ^16^

Most of the previous reports have studied the role of m6A modification on mRNA in tumors, but its m6A modification on lncRNA in tumors and tumor microenvironment remained at an unclear level. The purpose of this study was to explore the function of m6A‐related lncRNA in LUAD and TME based on public repositories. We obtained the transcriptome expression data and clinical information for patients with lung adenocarcinoma from The Cancer Genome Atlas (TCGA) and Gene Expression Omnibus (GEO) repository. Then, an 11 m6A‐related lncRNA signature was constructed after univariate independent prognostic regression analysis and least absolute shrinkage and selection operator (LASSO) analysis, and risk score of each individual was calculated independently. Our following findings demonstrated that the potential roles of signature composed of 11 lncRNA in the prognosis of lung adenocarcinoma.

## 2 METHOD AND MATERIALS

### 2.1 Datasets

Expression data of 594 LUAD patients were downloaded in the Cancer Genome Atlas (TCGA) repository, which including 59 normal samples and 535 tumor samples. Simple nucleotide variation data of 560 LUAD patients were downloaded from TCGA repository for TMB calculation. GSE31210 and GSE30219 acquired from Gene Expression Omnibus (GEO) repository were also used for signature construction and multi‐repository result validation. Sample with missing clinical information was removed during analyzing.

### 2.2 Bioinformatics

All statistical analyses were performed in R version 4.0.3. First of all, we selected 29 most common m6A regulators and conducted Spearman correlation analysis in TCGA and GSE31210 dataset, and m6A‐related lncRNA was screened by the standard correlation coefficient (|cor|) > 0.3. 1251 and 264 required lncRNA were acquired, respectively, in two databases. We found that the communal part in this lncRNA and the common 85 lncRNA were used for the signature construction. Then, after the univariate regression and LASSO analysis, prognostic model made up of 11 lncRNA was obtained. Kaplan‐Meier estimator was applied to draw the survival curves of single lncRNA or between two risk groups. Next, univariate, multivariate independent prognostic analysis, and ROC curve were used to evaluate the predictive efficiency and reliability of risk model. Estimation of Stromal and Immune cells in Malignant Tumor tissues using Expression data (ESTIMATE) is an algorithm to estimate the two major components of TME—stromal cells and immune cells—based on expression data, so that we utilize the method to calculate the stromalscore and the immunescore of each sample. After that, Wilcox test was applied to evaluate the infiltration between two risk groups. The consensus clustering method was applied to the new classification of LUAD, and the characteristics among the clusters were analyzed.

Then, we set our eyes on exploring the possible mechanism of m6A‐related lncRNA. We analyzed 3344 differentially expressed genes between risk groups using R package “limma.” GO, KEGG enrichment analysis, and metascape online tools were used to elucidate the signaling pathways and molecular mechanisms of possible DEGs enrichment. GSEA was used to explore pathways with significant enrichment in high‐risk groups. These biological pathways, cellular components, and molecular functions may reveal the mechanisms of m6A‐related lncRNA in LUAD.

### 2.3 Selection of m6A regulators

29 m6A regulators were chosen in this study. These m6A regulators are as follows: METTL3/5/14/16, YTHDF1‐3, YTHDC1/2, ALKBH5, FTO, WTAP, RBM15/15B/X, HNRNPC, HNRNPA2B1, VIRMA, IGF2BP1‐3, ZC3H13, SON, HAKAI, eIF3, ELAVL1, ABCF1, and ZCCHC4.^10^, ^12^, ^17^

## 3 RESULTS

### 3.1 Construction of independent prognostic model containing 11 lncRNA

**FIGURE 1:**
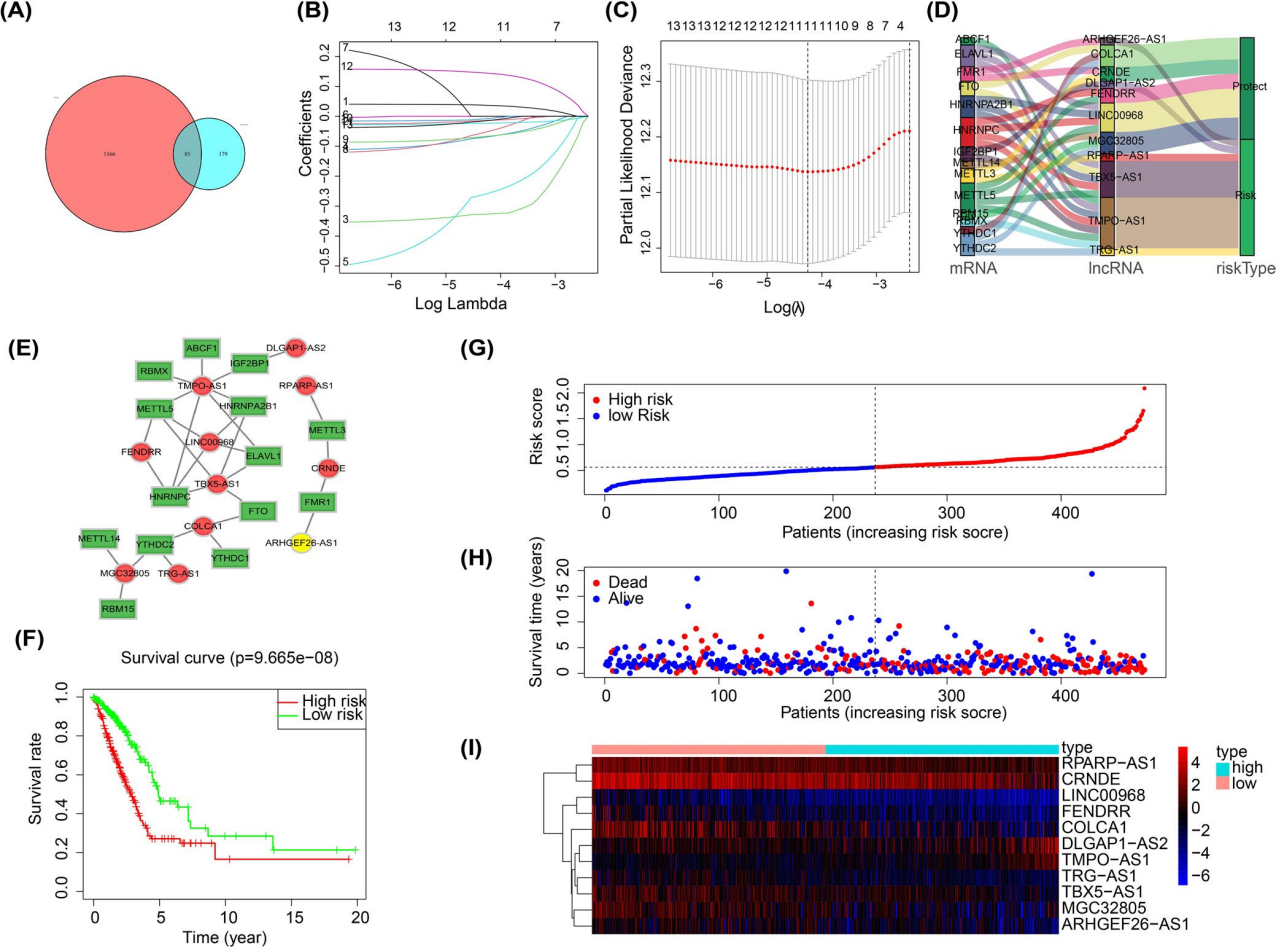
A, Common 85 m6A‐related lncRNA of 1251 in TCGA and 264 in GSE31210. B–C, LASSO analysis of 13 m6A‐related lncRNA that affected prognostic. D, Interactions between m6A regulators, m6A‐related lncRNA, and their function in LUAD. E, K–M survival curve of high‐ and low‐risk groups. F, Visualization of relationship between m6A regulators and m6A‐related lncRNA. G–I, Risk plot and heatmap of all samples

We download transcriptome data and clinical information of 594 LUAD patients from TCGA repository as the main body of bioinformatic analysis, and two datasets gained from GEO database (GSE30219 and GSE31210) as the supplement and validation part. Firstly, we performed Spearman correlation analysis for the purpose of finding m6A‐expression‐related lncRNA. According to the criterion that the |Spearman R| > 0.3 and results exist in both databases, we identified a total of 85 lncRNA for subsequent screening (Figure 1A). Then, after univariate regression analysis and LASSO analysis, we screened out 11 m6A‐related lncRNA that associated with prognostic for the construction of prediction signature (Figure 1B–C), and risk coefficients (coef value) were obtained of each lncRNA. The list of 11 lncRNA and coef value, respectively, is as shown in Table 1. The risk score of each sample was calculated by the formula: $\mathrm{Risk}\;\mathrm{score}=\sum_{j=1}^{n}\mathrm{Coef}j*ij$. According to the coef value of lncRNA, we can judge whether a lncRNA is a risk or protect lncRNA. Therefore, we constructed a Sankey diagram to depict the network of “m6A regulators—m6A‐related lncRNA—risk type” (Figure 1D), as well based on the correlation analysis, we visualized the relationship between m6A regulators and m6A‐related RNA by using cytoscape tool (Figure 1E). Next, we divided all samples of TCGA into high‐risk and low‐risk groups according to the risk score, and then used K–M survival curve to reflect the relationship between risk score and survival rate. As shown in Figure 1F, there was a significant difference between two groups (*P* = 9.665e−08). The risk curve and plot told us that the number of death cases increased as the increase in risk score distinctly (Figure 1G–I). These results indicated that risk score is a good predictor of overall survival rate in LUAD.

**TABLE 1:** Coefficient and Hazard Ratio of m6A‐related lncRNA

lncRNA	Coefficient	HR
DLGAP1‐AS2	0.036859	1.067096
COLCA1	−0.01392	0.934661
LINC00968	−0.32664	0.352524
MGC32805	−0.06904	0.829808
TRG‐AS1	−0.25897	0.66373
FENDRR	−0.05222	0.720177
RPARP‐AS1	−0.06707	0.85281
TBX5‐AS1	−0.00791	0.805625
CRNDE	−0.02293	0.953754
TMPO‐AS1	0.144436	1.301077
ARHGEF26‐AS1	−0.01469	0.790747

**FIGURE 2:**
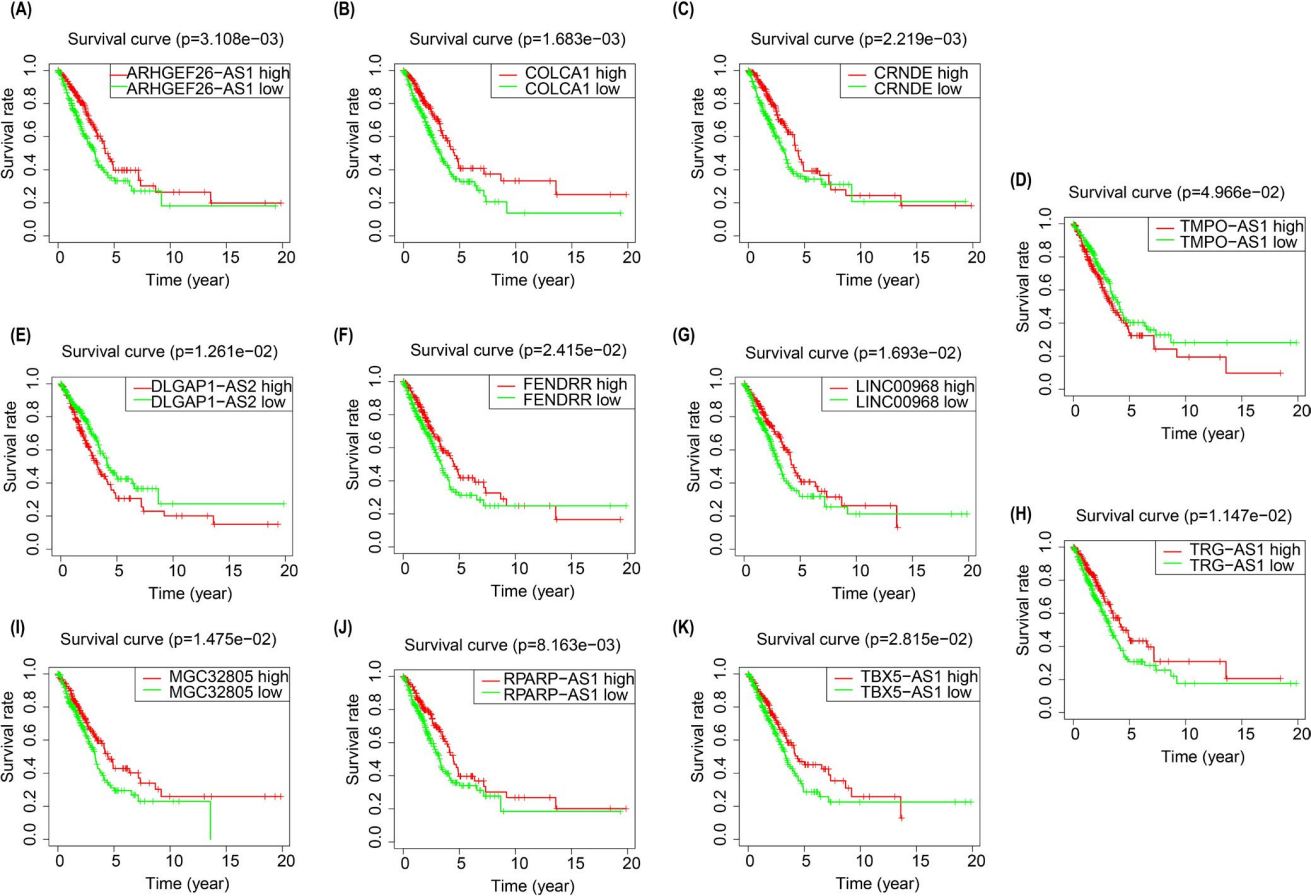
K–M survival curves between high and low expression of single lncRNA in m6A‐related lncRNA signature

Then, we plotted survival curves of each m6A‐related lncRNA with their grouping according to the expression level of the corresponding lncRNA. In the light of the results, the high expression of lncRNA TMPO‐AS1 and DLGAP1‐AS2 has a significant low overall survival rate, which is consistent with the coef value of all lncRNA. Survival between groups of all lncRNA has a significant difference (*P* < 0.05) (Figure 2).

### 3.2 Verification of the accuracy of the m6A‐related lncRNA signature

**FIGURE 3:**
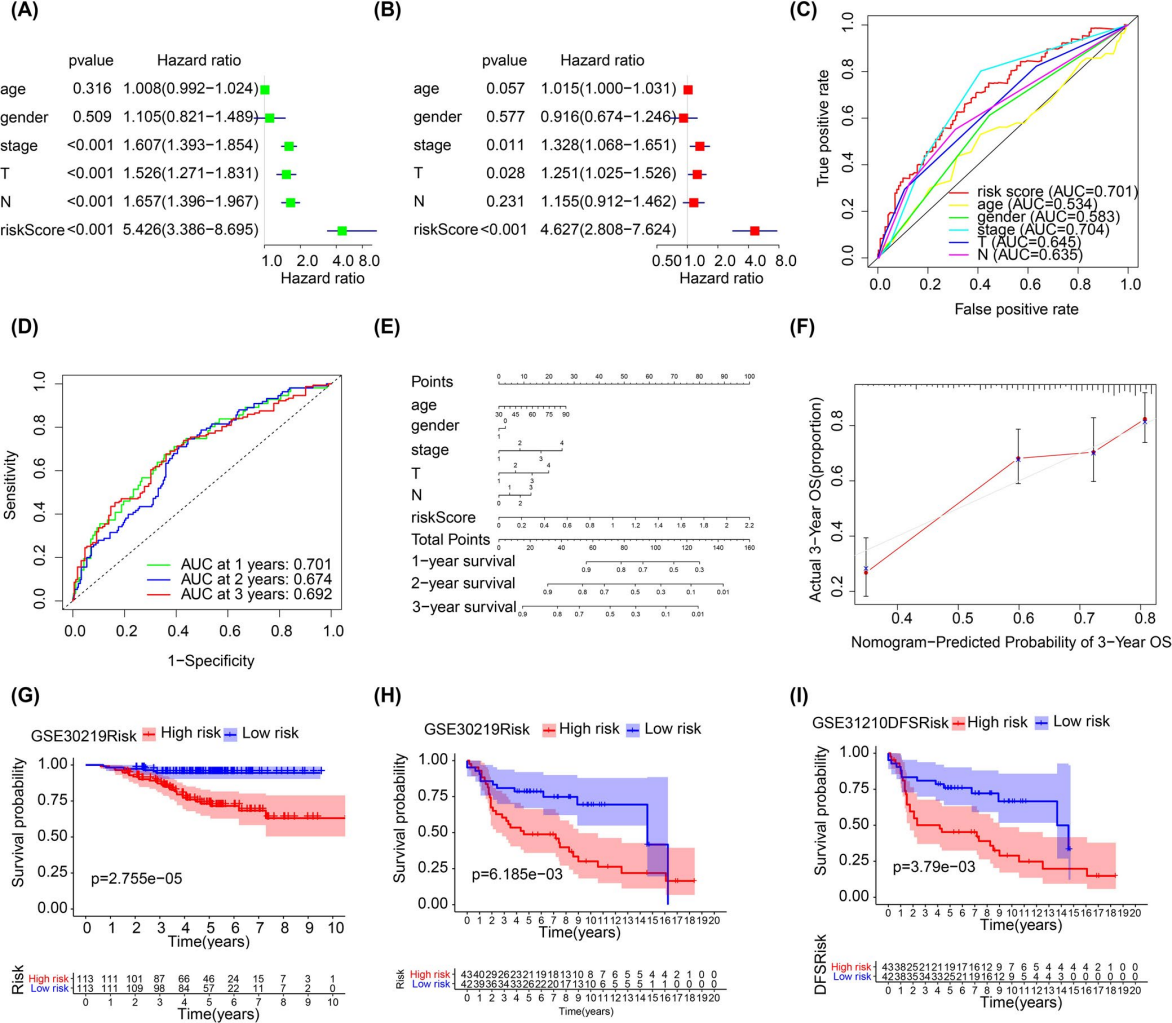
A, B, Univariate and multivariate prognostic analysis of risk score and other clinical characteristics. C, D, Multiple ROC curves of risk score in 1‐, 2‐, and 3‐years and AUG compared with age, gender, stage, T, N. E, F, Nomogram and calibration of m6A‐related lncRNA signature. G–I, Validation of survival across different risk score in external data sets, including OS and RFS

In this part, we used a variety method to verify the predictive reliability of this signature. Firstly, univariate and multivariate independent prognostic analysis were used to evaluate the predictive ability of risk score in TCGA cohort. It showed that risk score as a predictor has better predictive value than other clinical characteristics; these including age, gender, T stage, N stage, etc. (Figure 3A,B). Independent prognostic analysis of validation data set GSE30219 yielded the same results as shown in Figure S1, and multiple ROC curve confirmed this conclusion (Figure 3C). And the result of multi‐time ROC curve proved the effectiveness of risk signature in forecasting the 1‐, 2‐, and 3‐year survival of LUAD (Figure 3D). To combining the clinical characteristics and the risk signature, we constructed to establish a better clinical prognosis evaluation model, and we created a nomogram containing a serious of parameters and its calibration diagram (Figure 3E,F). Finally, we applied the risk model to the validation datasets and divided groups according to the risk score. We found that there were significant differences in overall survival (OS) and disease‐free survival (DFS) between the high‐ and low‐risk groups in the validation datasets GSE30219 and GSE31210 (Figure 3G–I). In conclusion, the results confirmed that the risk score has excellent predictive ability, which can be applied to all patients with LUAD.

### 3.3 Relationship between m6A‐related lncRNA and clinicopathological features

**FIGURE 4:**
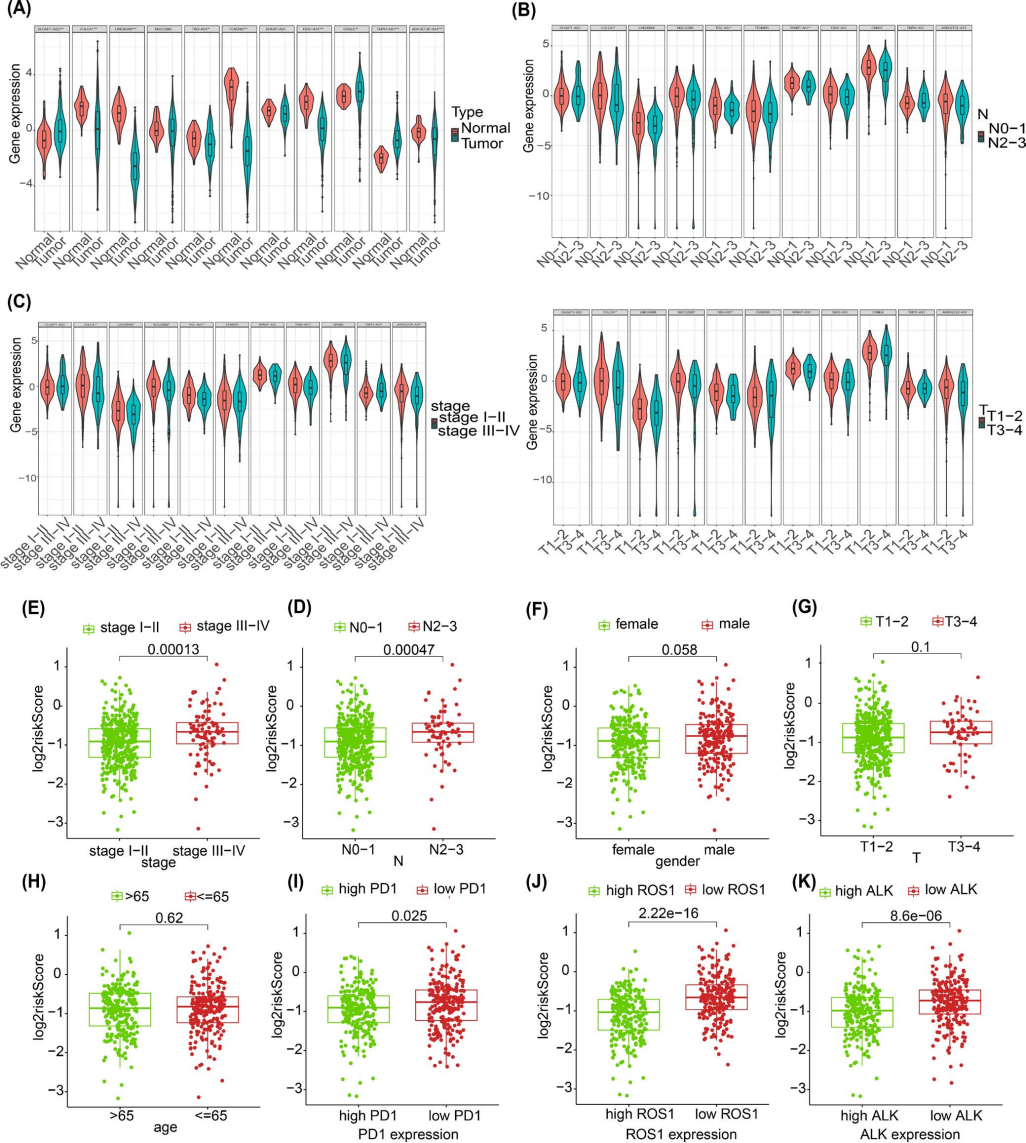
Correlation of 11 lncRNA signature and clinical features. A‐D, Expression of a single lncRNA in different tissue types, different lymph node conditions, different stages, and different tumor size. E–L, Relationship between risk score and clinical characteristics, immune checkpoints, ALK, and ROS1 gene

In TCGA cohort, we compared the distribution of age, gender, and other clinical characteristics between high‐ and low‐risk groups, and Student's *t* test was performed to analyze the differences between groups. The output results were shown in Table 2. The results manifested that the m6A‐related lncRNA risk signature was significantly correlated with gender, stage, and lymph node metastasis status. Then. we explored association between clinical characteristics and m6A‐related lncRNA. The expression of almost all lncRNA was significantly different between tumor and normal groups, except MGC32805 (Figure 4A). Half of this lncRNA expression was significantly correlated with lymph node metastasis (Figure 4B), and most of these lncRNA expressions in late stage (stage III‐IV) were significantly different from early (stage I‐II) (Figure 4C), and the expression levels of two lncRNA (COLCA1 and TRG‐AS1) were associated with T stage (Figure 4D). In the next exploration of risk score and clinical characteristics, m6A‐related lncRNA signature risk score was significantly associated with lymph node metastasis and patient grade (Figure 4E–D). There was also some association between risk score and gender, but not significant enough (Figure 4F). However, there no significant correlations between risk score and age/tumor size (Figure 4G–H). These findings supported to some extent by the results in Table 2. We also evaluated the role of m6A‐related lncRNA in important immune checkpoints and frequently altered genes in LUAD. The results showed that individuals with low expression of PDCD1 gene (PD‐1 checkpoint), ROS1 gene, and ALK gene and had higher risk score (Figure 4I–L). All these results suggested that m6A‐related lncRNA may have an effect on the lymph node metastasis of tumor, high risk and later stage are closely related usually. In addition, m6A‐related lncRNA may play an unclear role in immune checkpoint PD‐1 and ALK genes.

**TABLE 2:** Correlation between riskscore and clinical features (*P value <0.05, ** *P* value <0.01, *** *P* value <0.005, **** *P* value <0.001)

Clinical	Group	*n*	Mean	SD	*t*	*P* value
Age	<=65	227	0.603	0.269	1.031389	0.303
	>65	246	0.578	0.243		
Gender	female	252	0.566	0.232	−2.16333	0.031^*^
	male	221	0.617	0.279		
Stage	stage I–II	372	0.566	0.237	−3.44732	0.001^***^
	stage III–IV	101	0.677	0.3		
T	T1‐2	411	0.585	0.257	−1.1459	0.255
	T3‐4	62	0.624	0.248		
N	N0‐1	402	0.573	0.241	−2.92134	0.004^***^
	N2‐3	71	0.686	0.312		

### 3.4 M6A‐related lncRNA can influence TME of LUAD

**FIGURE 5:**
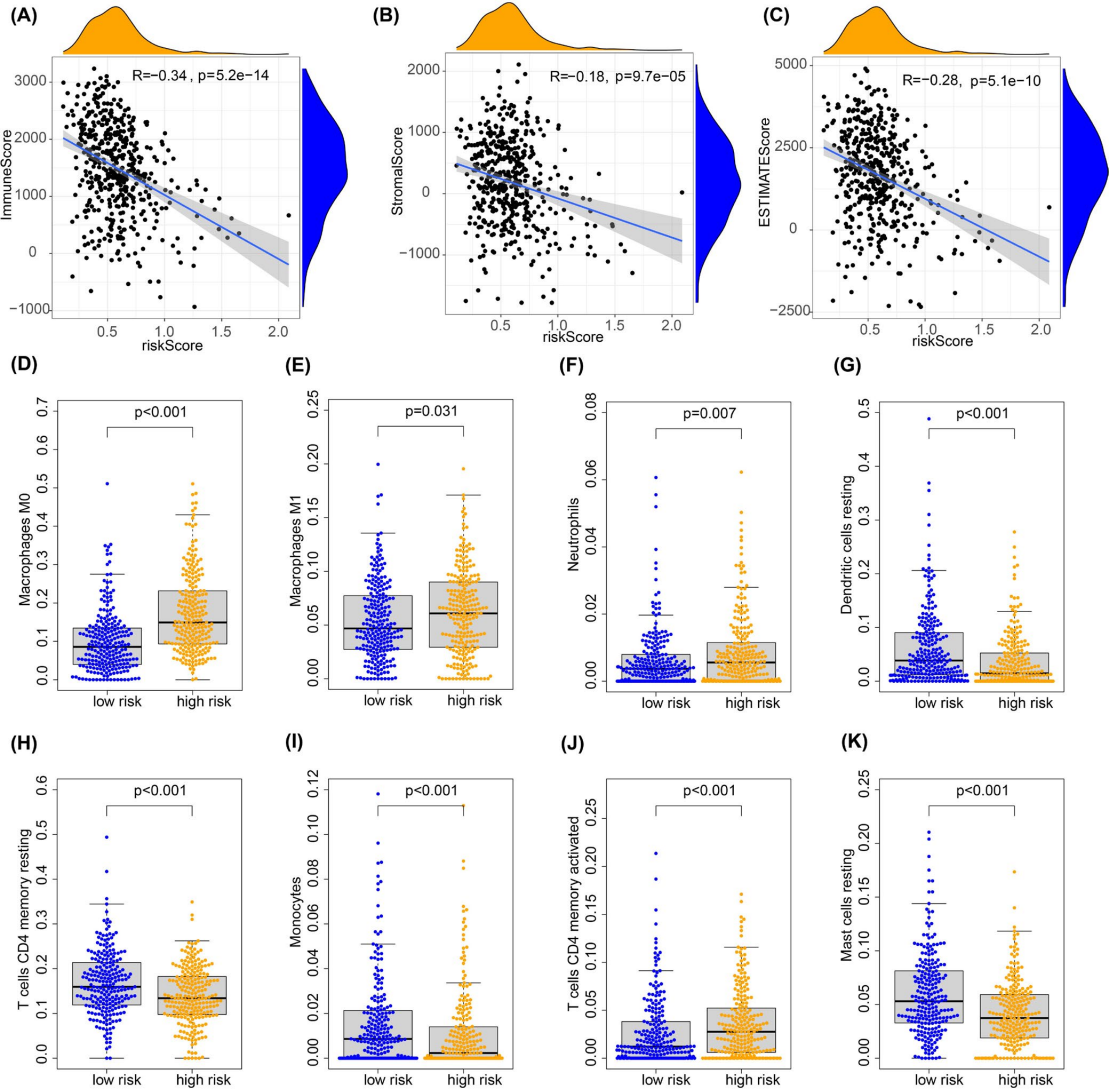
M6A‐related lncRNA and TME. A–C, A positive correlation between tumor purity (content of stromal cell and immune cell) and risk score. D–K, The degree of infiltrations of various immune cells in TME between different risk groups

We believed that m6A‐related lncRNA may affect TME. In order to explore the specific part of this effect, we used the ESTIMATE algorithm to estimate the purity of tumor tissue of 594 patients, and the Spearman correlation analysis was carried on between the risk score and the calculated score (stromalscore, immunescore, and ESTIMATE score). Results showed that the three calculated scores decline prominently as the risk score increased (Figure 5A–C). This suggests that higher tumor purity means a higher prognostic risk for the patient, as the amounts of immune cells and stromal cells in the TME of the patient were reduced.

**FIGURE 6:**
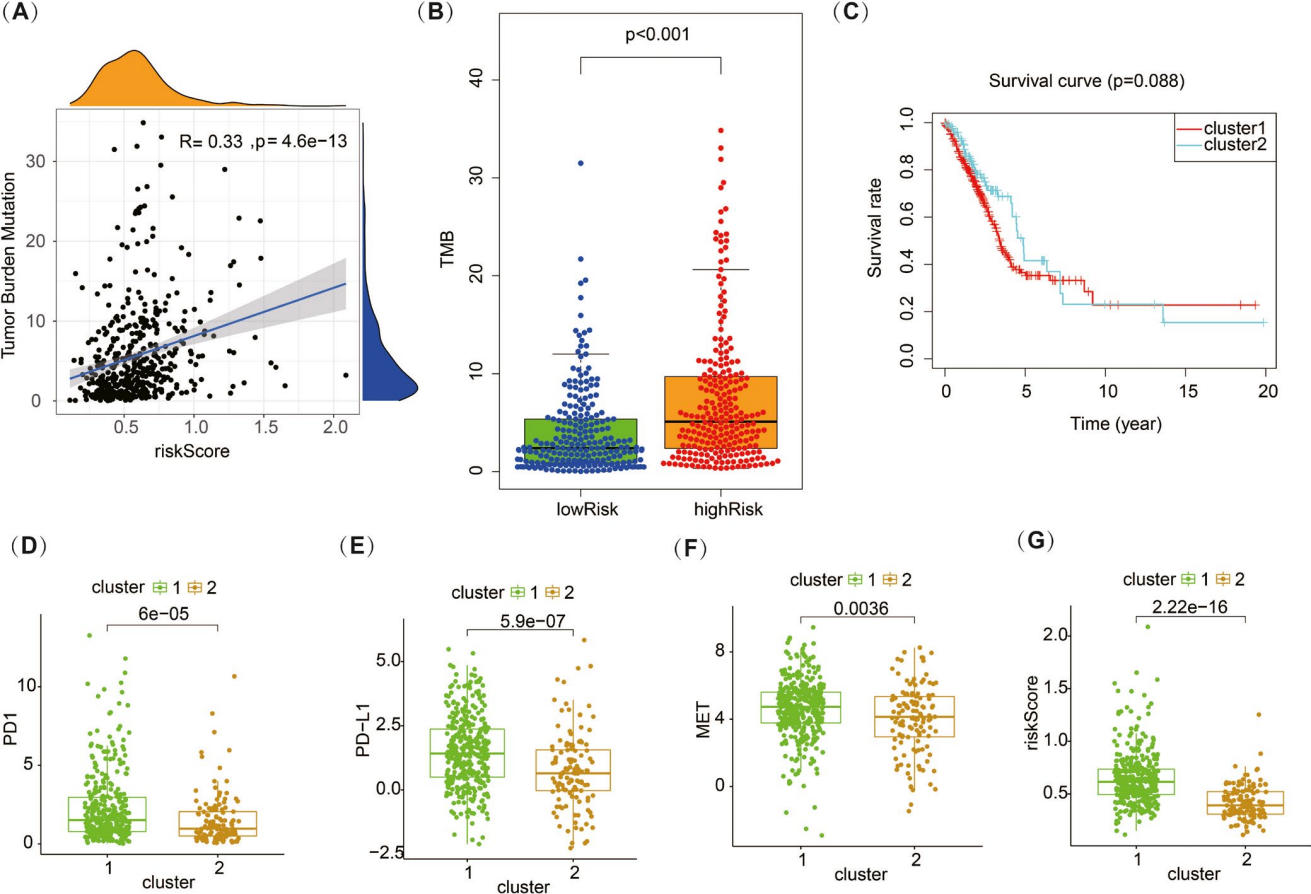
A, Correlations of TMB and risk score. B, Significant difference in TMB between high‐ and low‐risk groups. C, Overall survival curve between cluster s1 and 2. D–G, Different immune checkpoints, common driver genes of lung cancer

To further investigate the infiltration of immune cells in the TME, we used the CIBERSORT tool to calculate the relative abundance of various immune cells according to the transcriptome of all samples. Based on the result of CIBERSORT, we explored the effect of the m6A‐related lncRNA signature on the infiltration of the immune cells according to the groups of high and low risk. We found that the TME in the high‐risk group showed significant enrichment infiltration of macrophages and neutrophils (Figure 5D–F). Infiltration of dendritic cells, mast cells, and monocytes predicts low‐risk and good prognosis (Figure 5G–I). With respect to T‐cell infiltration, we found that CD4+ memory T cells had a significant tendency to be activated with the increase in risk (Figure 5J,K). We also calculated TMB in lung cancer patients and analyzed the associated with risk score. The results showed that TMB was positively correlated with risk score (Figure 6A), and the difference was significant between high‐ and low‐risk groups (Figure 6B). All these results suggested that m6A‐related lncRNA affects the prognostic of patients by changing the TME and the infiltration of immune cells.

### 3.5 Analysis and exploration of subtypes based on the m6A‐related lncRNA

We explored a new subtype classifying method of LUAD using the consensus clustering method, and all the samples were divided into two clusters in this way (Figure S2). We analyzed overall survival rates between two clusters and found no significant differences (Figure 6C). Analysis of the expression levels of common immune checkpoints (PD1 and PD‐L1 in our study) and lung cancer driver genes (ALK, EGFR, ROS1, and MET in our study) between two clusters revealed significant differences in PD1, PD‐L1, and MET gene expression (Figure 6D–F). There was also a significant difference in risk scores between the two clusters (Figure 6G).

### 3.6 Exploration on the mechanism of m6A‐related lncRNA in LUAD

**FIGURE 7:**
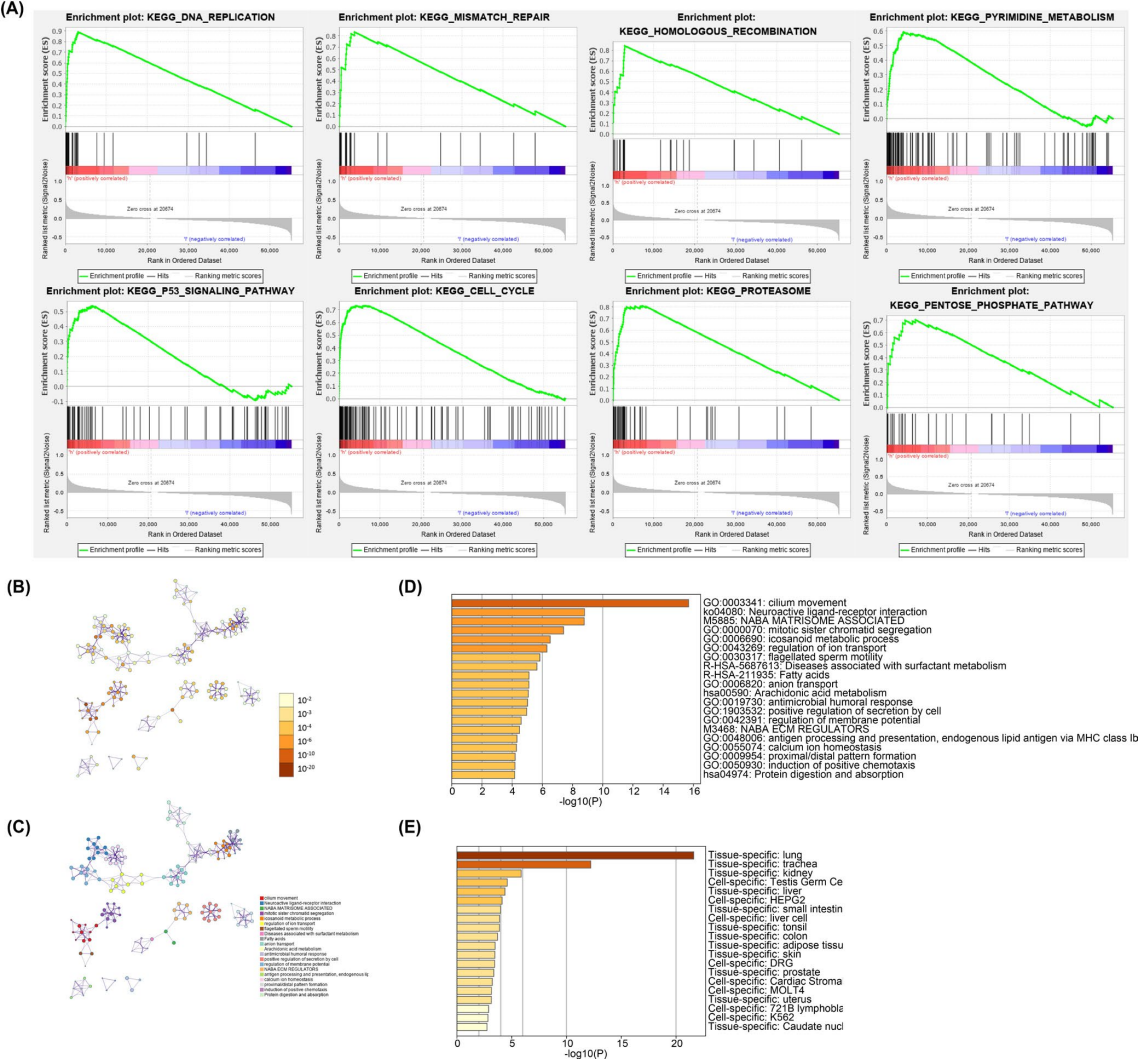
A, GSEA analysis between two risk groups. B–D, Metascape on analysis signaling pathways of DEGs. E, Tissue and cell line specific of DEGs

In order to explore the specific mechanism of m6A‐related genes affecting LUAD, we used GSEA software to analyze the signal pathways enriched in high‐ and low‐risk group, respectively. The screening criteria of results are |NES|>1, NOM pval < 0.05, and FDR < 0.25. GSEA tests run 1000 times. We then listed 8 pathways that were most significantly enriched in the high‐risk group, which were found to be most related to the cell cycle (Figure 7A). Therefore, we speculate that m6A‐related lncRNA promotes tumorigenesis by influencing metabolic pathways in the cell cycle. Next, we looked for different expression genes (DEGs) between the high‐ and low‐risk groups, and a total of 3,344 DEGs were found (Figure S3). Metascape (http://metascape.org/) online tool and R software were applied to explore the enrichment of DEGs pathways. The results of metascape software showed that the most enriched signaling pathways were cilium movement, neuronal activity ligand‐receptor interaction, and NABA matrisome associated (Figure 7B–D). The result was also confirmed by GO and KEGG enrichment analysis (Figure S4). Metascape tool also told us that the lung is the most specific tissue of these genes (Figure 7E).

**FIGURE 8:**
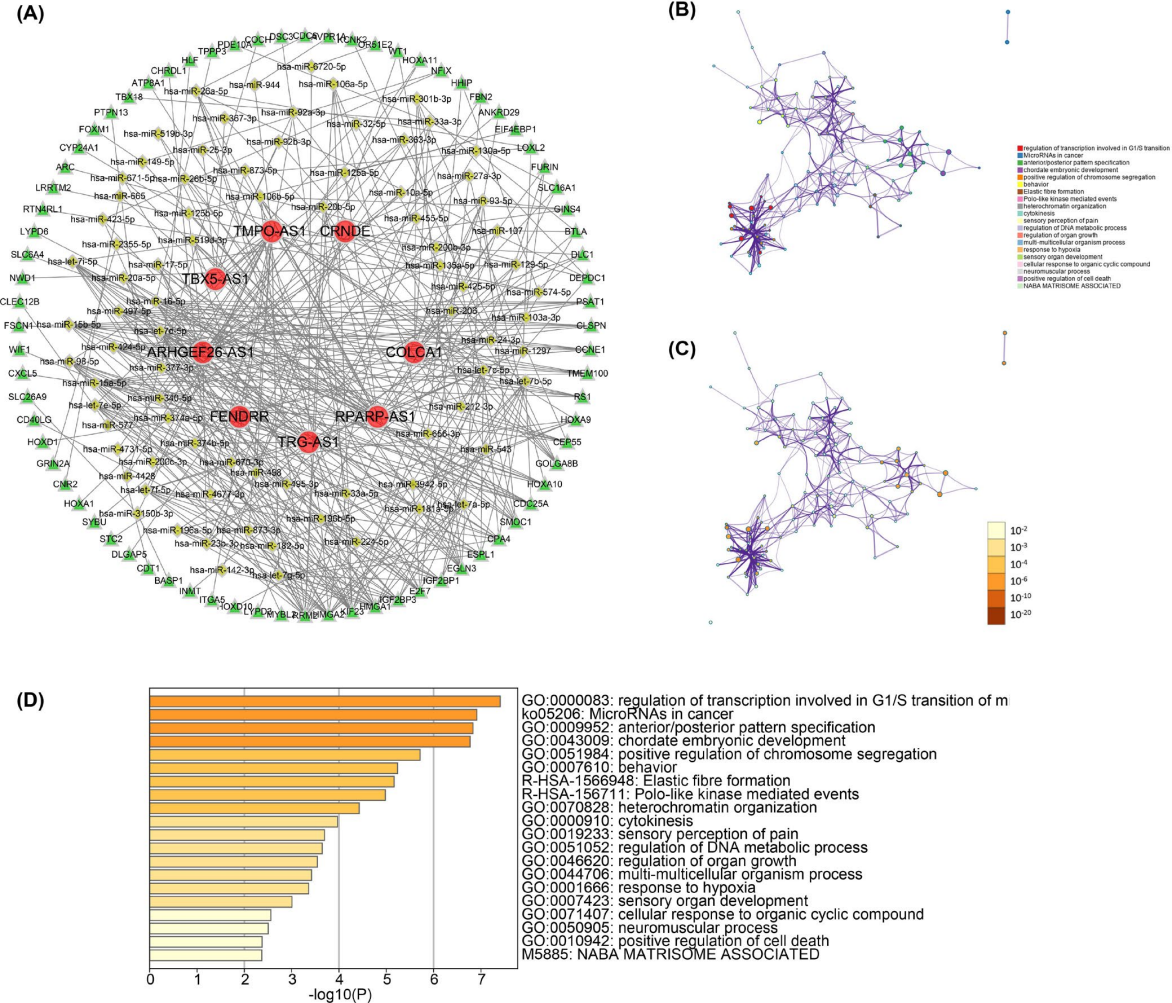
A, Construction and visualize of ceRNA network of 11 m6A‐related lncRNA. B–D, Signaling pathways enrichment analysis of target mRNA of m6A‐related lncRNA on Metascape tool

In the end, we constructed the ceRNA network of 11 lncRNA included in the signature to find out the upstream and downstream regulation factors of m6A‐related lncRNA. The Starbase and miRcode databases were used to search for the upstream miRNA of lncRNA. The target genes of miRNA were found in TargrtScan, mirTarbase, and miRDB databases, and the records in all three databases were used as the selection criteria. We finally got 82 miRNA and 76mRNA, and cytoscape software was used to visualize the interactions of them (Figure 8A). The signaling pathway enrichment analysis of these mRNA showed that m6A‐related lncRNA may be involved in the regulation of DNA transcription and metabolism in the G1/S phase, and also related to miRNA in the tumor (Figure 8B–D). Finally, we analyzed the protein‐protein interactions of 76 mRNA in online tool String (https://string‐db.org/) (Figure S5).

## 4 DISCUSSION

Lung cancer is a major problem in the development of human medicine. The difficulty of early detection and diagnosis causes only 16% survival rate of lung cancer patients in China.^18^ Therefore, it is a potential value to search for new serological or histological prognostic markers for the diagnosis, prognosis, and treatment.

As the most abundant chemical modification, N‐6 methylation plays an important role in many biological processes, especially in human cancer.^19^ A study by Jinyan W et al. indicate that demethylase ALKBH5 was involved in autophagy, hypoxia, and other processes, and its disorder could regulate the occurrence of human tumor^20^; Tao G et al. demonstrated ALKBH5 can promote tumor progression by enhancing the demethylation in colon cells.^21^ In recent years, other studies also confirmed the function of m6A regulators in malignant tumors through all human systems. For example, ALKBH5 promotes the invasion of lung adenocarcinoma through FOXM1 signaling pathway^22^; while in the liver, high expression of ALKBH5 inhibits the malignancy of hepatocellular carcinoma.^23^ Although METTL3 binds to promoters to promote proliferation of tumor cells in acute myeloid leukemia,^24^, ^25^ increased expression often leads to apoptosis of tumor cells in triple‐negative breast cancer.^26^ N‐6 methylation on other RNA has also been studied for the past few years. It has been found that m6A modification on circRNA can promote tumor development by driving circRNA translation, affecting binding to RBP, or changing the methylation of downstream targets of circRNA.^27^, ^28^, ^29^ Several reports have also verified that lncRNA is also the target of m6A affecting the tumor process. For example, GATA3‐AS1 promotes the metastasis of liver cancer, and the downregulation of tumor suppressor gene GAS5 in cervical cancer is all inseparable from the shadow of m6A.^11^, ^30^, ^31^ LncRNA is also very important in the progression of lung cancer. Some studies have shown that LncRNA can participate in transcriptional regulation in non‐small cell lung cancer and enhance tumor tolerance to chemotherapy.^32^, ^33^, ^34^

It is important to note that the same m6A regulators may play a different role in different tissues. Therefore, the objective of this study was to explore the function of m6A‐related lncRNA in LUAD. In order to achieve this goal, we downloaded data from TCGA and GEO repositories for lncRNA screening and signature establishment. Using Spearman analysis to get lncRNA closely related to m6A, and so as to make the signature more reliable, we extracted the common part of the two data sets. Univariate regression analysis was used for the screening of prognostic m6A‐related lncRNA, and a signature composed of 11 lncRNA is obtained through LASSO. We found that the prognosis and patient's risk score according to signature are significantly related. Later, after independent prognostic analysis, ROC curve, and external datasets verification, we thought that the ability of m6A‐related lncRNA signature to predict patient prognosis is trustworthy. The m6A‐related lncRNA is also having a bearing on clinical traits, for instance, late‐stage (stage III–IV) and lymph node metastasis showed a higher risk score. It is worth mentioning that the low expression of immune checkpoint PD1 and ALK genes also presumed a higher risk score in LUAD. After exploring the components of TME, we found that the signature is significantly having relations of tumor purity and immune cell infiltration abundance. High purity tumors indicate a high‐risk and a poor prognosis, as well the activation of CD4+ memory T cells and the infiltration of macrophages also meant an increased risk of prognosis. In the later study of the mechanism of m6A‐related lncRNA in LUAD, we used GSEA, GO, and KEGG to get different pathway enrichment between high‐ and low‐risk groups. The results indicated that the signal pathways related to cell cycle are more active in the high‐risk group, while most of the DEGs are related to the ciliary movement and neuroactive ligand‐receptors interaction. Finally, in the construction of ceRNA network, we found the upstream and downstream regulatory factors of lncRNA, and we discovered that the target mRNA of m6A‐related lncRNA may affect the progression of tumor by regulating the DNA transcription of G1/S phase of cells.

In conclusion, m6A‐related lncRNA is a potential diagnostic target and plays an important role in the treatment and prognosis in LUAD.

## CONFLICT OF INTEREST

The authors declare that there is no relative conflict of interest.

## AUTHOR CONTRIBUTIONS

Jiang Zheng, Zhuochen Zhao, and Junhu Wan jointly responsible for the data acquisition and analysis. Jian Zheng, Yangxia Wang, Zhengwu Yang, and Zhuofang Li involved in the production of the Figures and Tables. Zhuochen Zhao and Manman Guo conceived and wrote the article. Liang Ming and Zhaobing Qin approved the final study. Jian Zheng and Zhuochen Zhao contributed equally to this work.

## CONSENT FOR PUBLICATION

Written informed consent for publication was obtained from all participants.

## Supporting information

Figure S1

Figure S2

Figure S3

Figure S4

Figure S5

## Data Availability

All the data in this study are download from public databases as described in the passage. Data could be acquired from TCGA (https://portal.gdc.cancer.gov/) and GEO (https://www.ncbi.nlm.nih.gov/) repositories.
