# Supplementary figures and images for: N‐6 methylation‐related lncRNA is potential signature in lung adenocarcinoma and influences tumor microenvironment
Source: J Clin Lab Anal. 2021 Sep 24;35(11):e23951. doi: 10.1002/jcla.23951 (PMC8605119; doi:10.1002/jcla.23951)

Figure S1. Univariate and multivariate prognostic analysis of risk score in GSE30219.


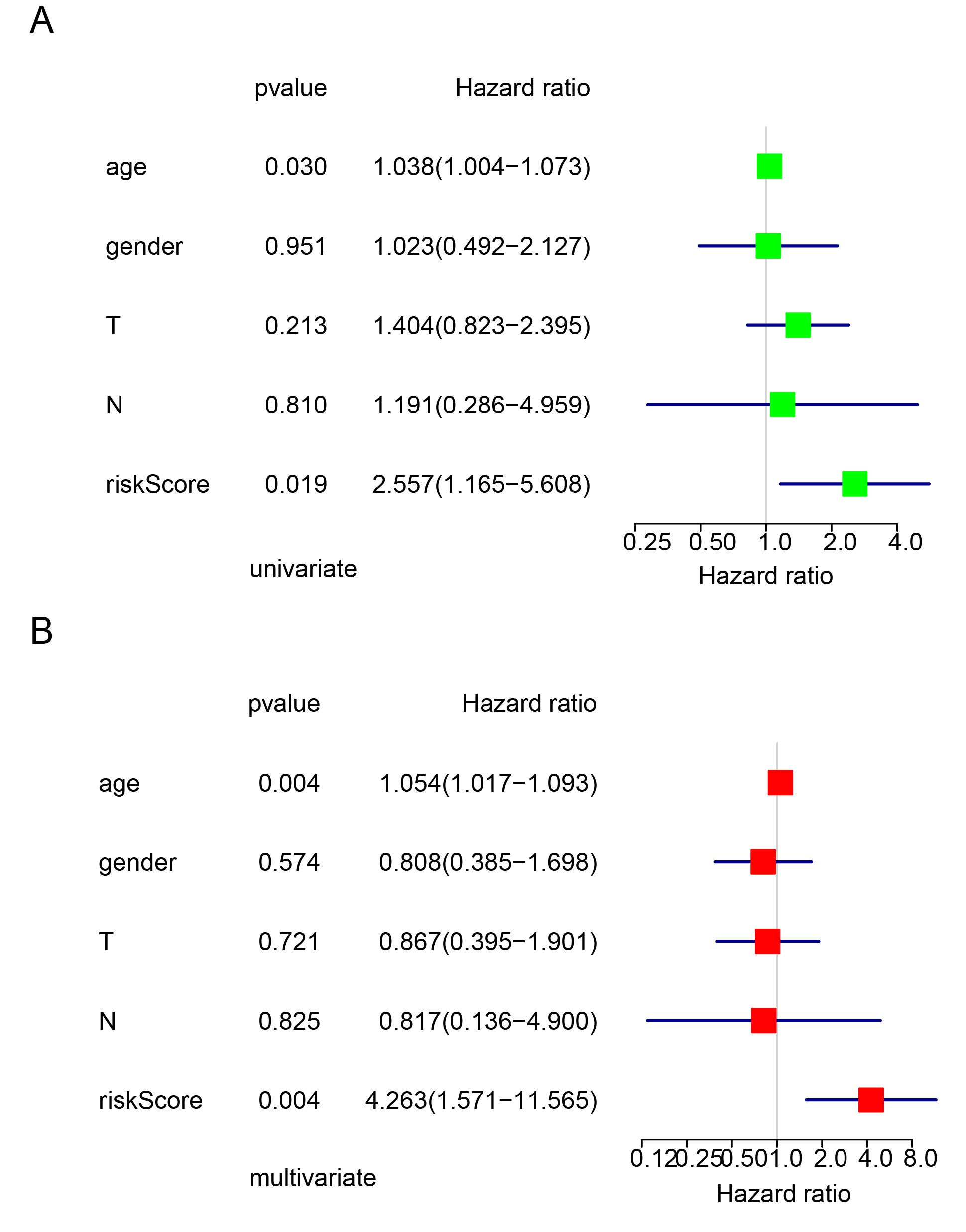

Supplement: Supplementary file 1 — Figure S1 [file JCLA-35-e23951-s004.docx]

Figure S2. Consensus Cluster of m6A-related lncRNA.


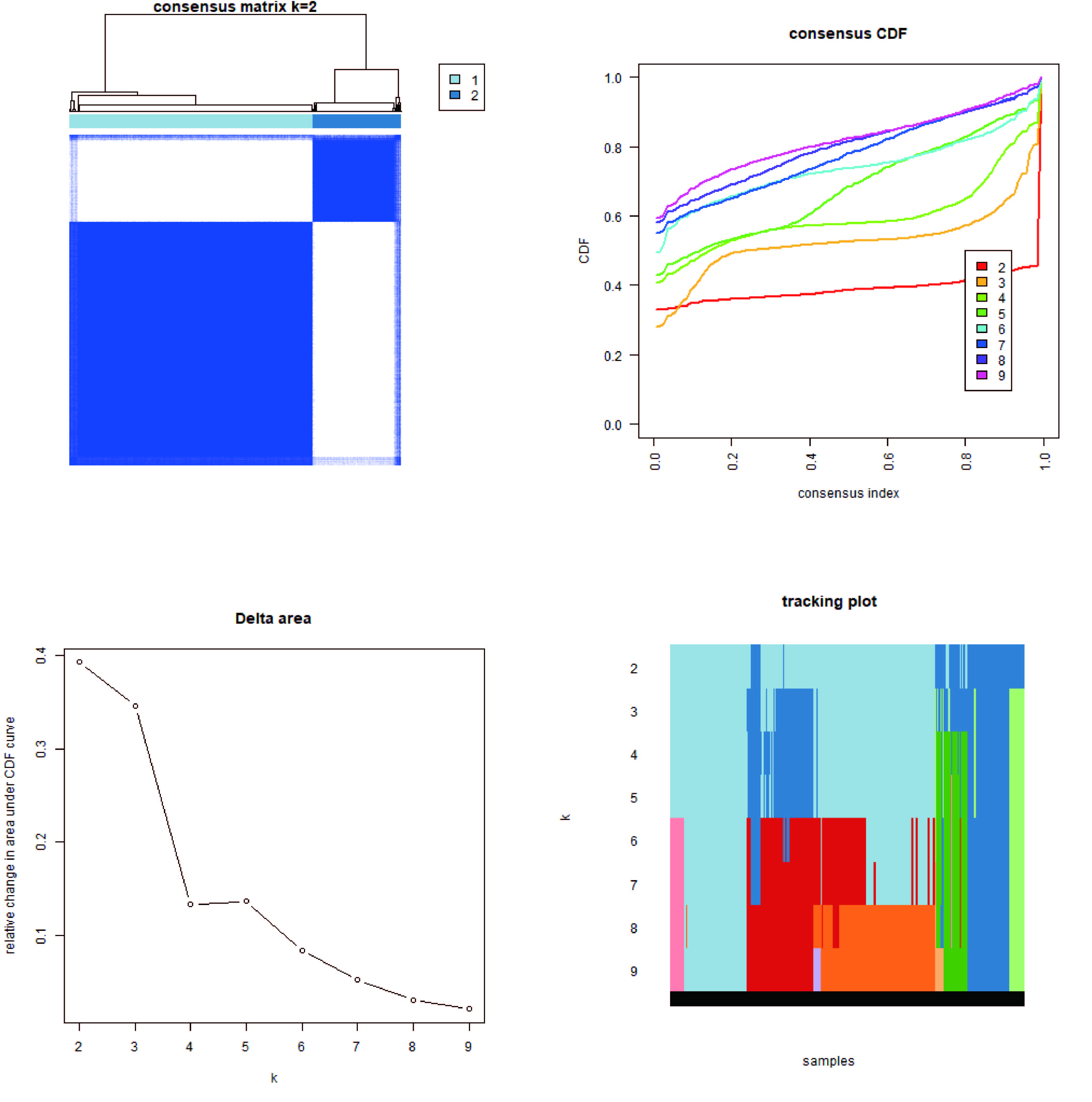

Supplement: Supplementary file 2 — Figure S2 [file JCLA-35-e23951-s001.docx]

Figure S3. Volcano plot and heatmap of DEGs between high and low groups.


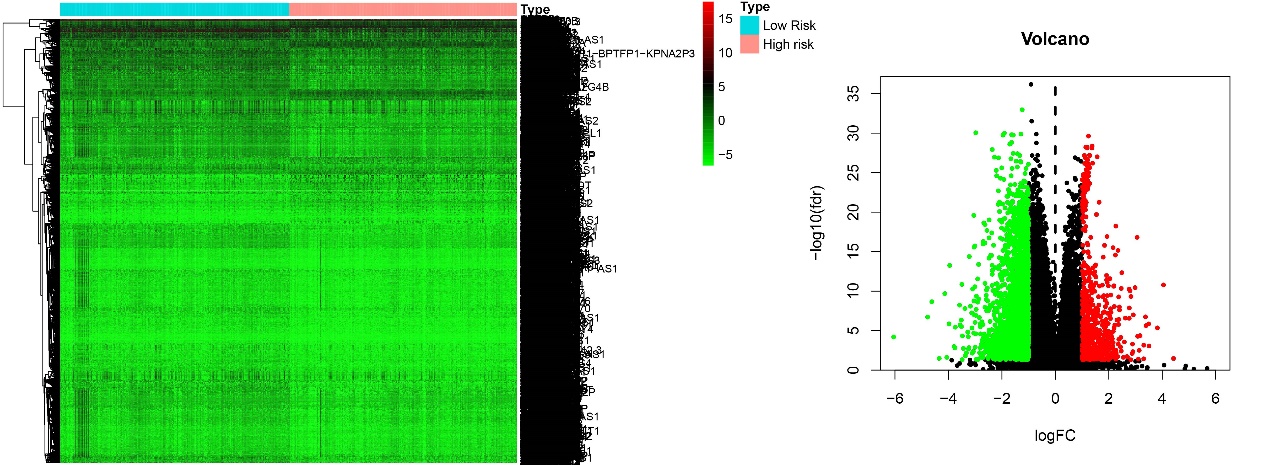

Supplement: Supplementary file 3 — Figure S3 [file JCLA-35-e23951-s005.docx]

Figure S4. GO and KEGG analysis of DEGs performed in R.


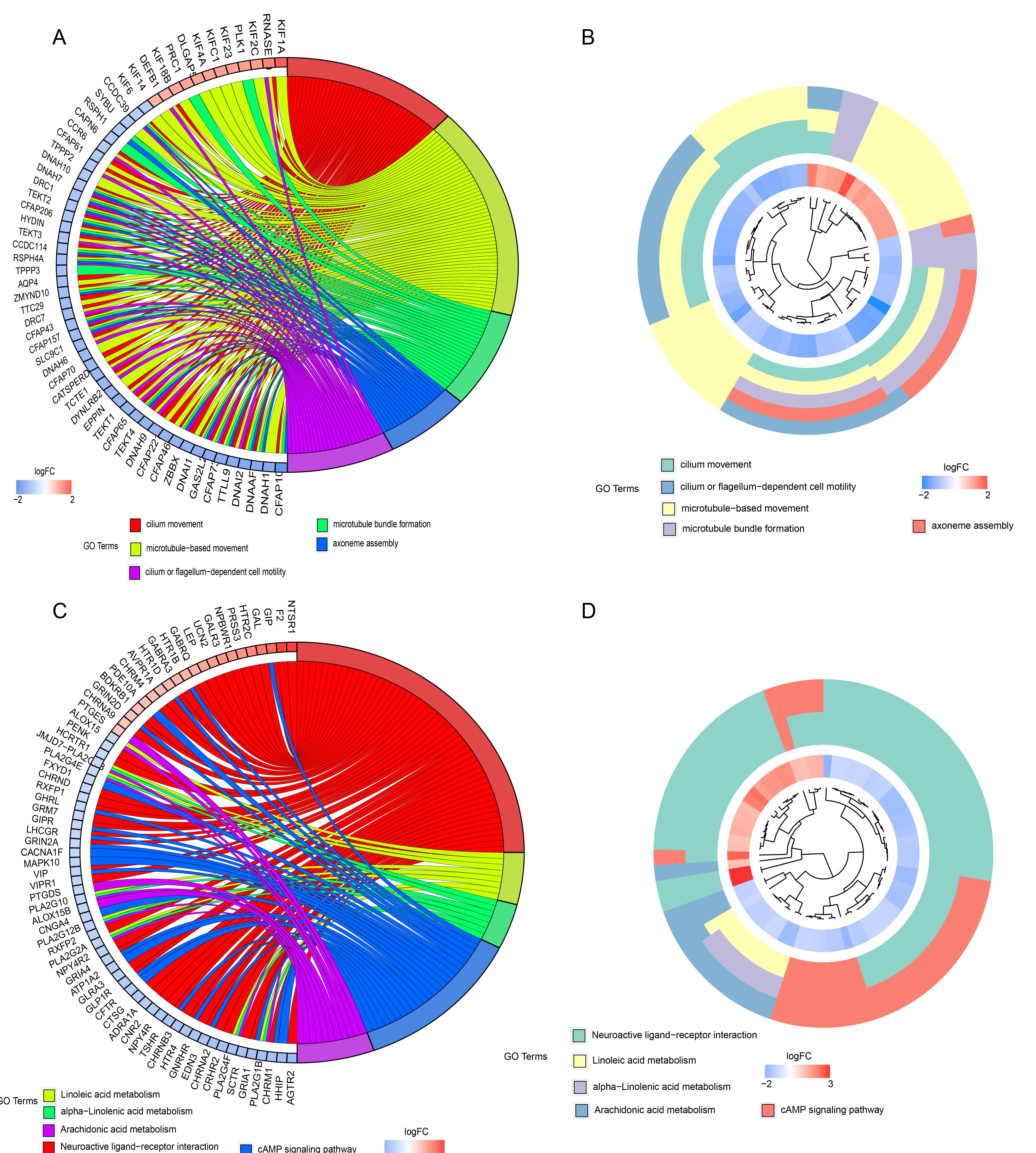

Supplement: Supplementary file 4 — Figure S4 [file JCLA-35-e23951-s003.docx]

Figure S5. Protein-protein interactions diagram of target mRNA of 11 m6A-related lncRNA.


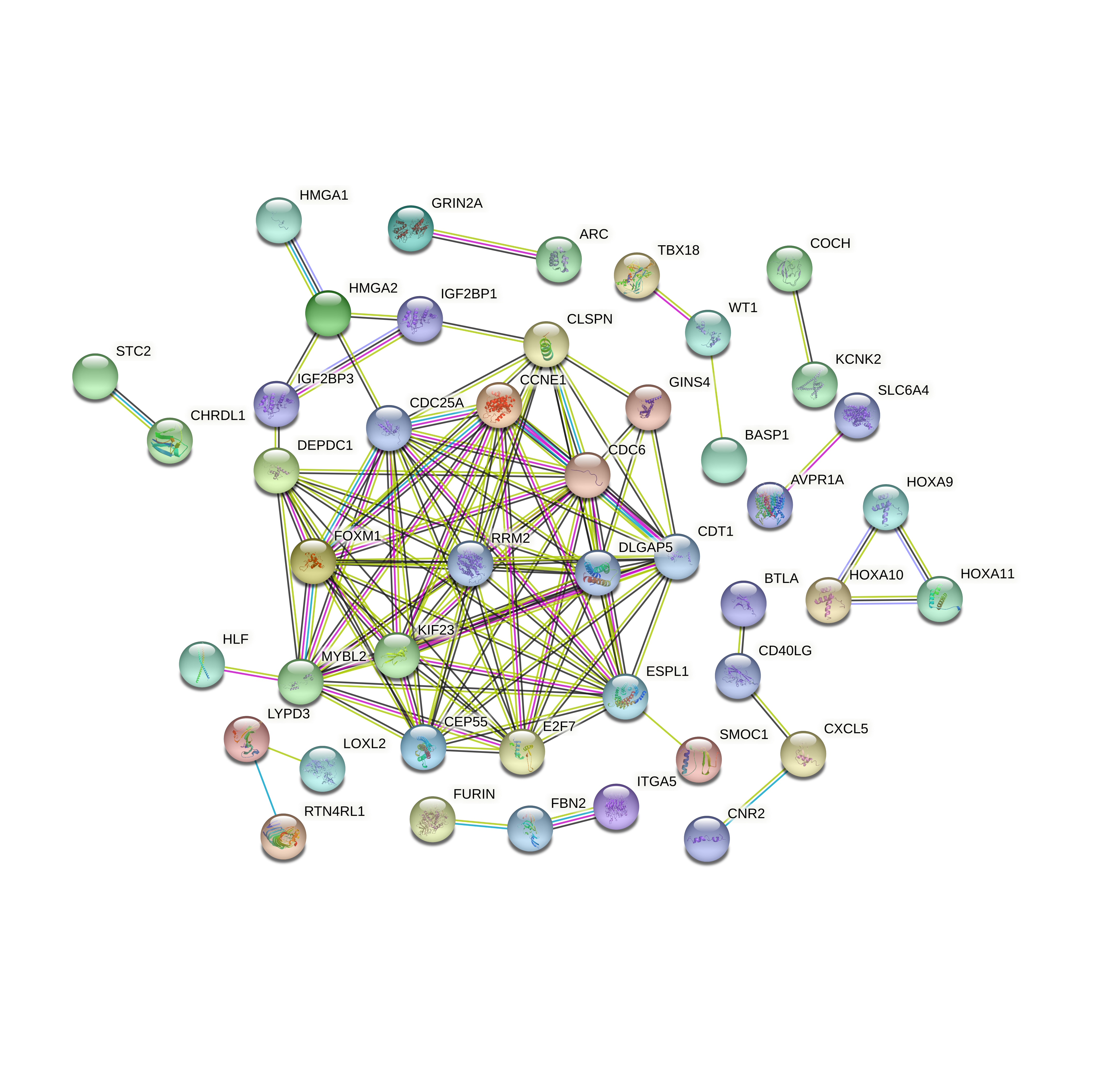

Supplement: Supplementary file 5 — Figure S5 [file JCLA-35-e23951-s002.docx]
